# Supplementary material for: Structural and biochemical evidence supporting poly ADP-ribosylation in the bacterium Deinococcus radiodurans
Source: Nat Commun. 2019 Apr 2;10:1491. doi: 10.1038/s41467-019-09153-6 (PMC6445106; doi:10.1038/s41467-019-09153-6)
Supplement: Supplementary file 1 — Supplementary Information [file 41467_2019_9153_MOESM1_ESM.pdf]

Structural and biochemical evidence supporting poly ADP-ribosylation in the bacterium *Deinococcus radiodurans*

Cho et al.

Supplementary Table 1. Selected BLAST hits of DrPARG bearing the PARG signature motif.

Protein accession No. / PDB code	Organism	Length (a.a.)	Sequence identity to DrPARG	Sequence similarity to DrPARG	Residue corresponding to Asp260 of DrPARG	Residue corresponding to Thr267 of DrPARG
PTA68839	*Deinococcus sp.*	279	58.39	66.43	Asp	Val
ADV68331	*Deinococcus maricopensis*	278	46.98	57.05	Asp	Thr
EEF58666	*Pedosphaera parvula*	277	46.42	54.27	Asp	Thr
APR75618	*Minicystis rosea*	269	45.70	57.39	Asp	Asn
ABF86196	*Myxococcus xanthus*	271	45.33	56.40	Glu	Asn
ATB50252	*Myxococcus macrosporus*	271	44.98	56.06	Glu	Asn
AFE05063	*Corallococcus coralloides*	273	44.83	55.86	Glu	Asn
KPC60454	*Streptomyces chattanoogensis*	275	44.79	55.56	Asp	Thr
KYF85377	*Sorangium cellulosum*	275	44.67	55.67	Asp	Asn
AEI65031	*Myxococcus fulvus*	271	44.64	55.71	Glu	Asn
SNR91617	*Actinomadura mexicana*	276	44.63	53.02	Asp	Arg
EDY20783	*Chthoniobacter flavus*	269	44.52	52.74	Asp	Val
EPX64350	*Cystobacter fuscus*	271	44.48	54.14	Glu	Asn
OKL52149	*Actinomyces hordeovulneris*	268	44.07	55.25	Asp	Thr

3SIG_A	*Thermomonospora curvata*	277	43.77	52.53	Asp	Arg
PQO41191	*Blastopirellula marina*	279	43.69	54.95	Asp	Ile
RBP39074	*Roseimicrobium gellanilyticum*	280	43.69	54.61	Asp	Val
PKK14663	*Thermomonospora sp.*	279	43.62	52.68	Asp	Arg
SNS65478	*Actinomadura meyerae*	271	43.58	51.01	Asp	Arg
OJW24701	*Planctomycetales bacterium*	279	42.86	54.82	Asp	Thr
ADD43214	*Stackebrandtia nassauensis*	270	42.81	55.14	Asp	Val
KUN49339	*Streptomyces olivochromogenes*	282	42.76	56.23	Asp	Thr
OLT31452	*Actinomadura sp.*	276	42.66	53.92	Asp	Arg
AFR06340	*Nocardiopsis alba*	269	42.66	54.27	Asp	Val
EKX62864	*Streptomyces ipomoeae*	270	42.61	55.33	Asp	Val
OEJ73421	*Desertifilum sp.*	278	42.52	56.80	Asp	Ile
SNR60489	*Hymenobacter mucosus*	283	42.52	54.42	Asp	Val
KOX22736	*Streptomyces sp.*	276	42.52	52.72	Asp	Thr
SOR77333	*Streptomyces chartreusis*	281	42.00	52.67	Asp	Val
EGE40190	*Streptomyces sp.*	273	41.44	53.42	Asp	Ala
PQO40474	*Blastopirellula marina*	279	41.30	54.27	Asp	Ile

GAT67893	*Planomonospora sphaerica*	299	41.10	52.43	Asp	Thr
RIH85454	*Meiothermus terrae*	282	40.94	54.03	Asp	Thr
BAZ14810	*Calothrix sp.*	277	40.82	52.72	Asp	Thr
PIG17387	*Streptomyces sp.*	299	40.51	53.05	Asp	Thr
PSL29762	*Chitinophaga ginsengisoli*	289	40.47	51.84	Asp	Thr
OLO29462	*Streptomyces sp.*	273	40.41	53.08	Asp	Val
ARF65628	*Streptomyces violaceoruber*	273	40.41	52.40	Asp	Val
RGC65481	*Micromonospora sp.*	280	40.40	52.53	Asp	Thr
OQX00738	*Thiothrix lacustris*	270	40.27	51.54	Ala	Asn
EAY25794	*Microscilla marina*	289	39.67	54.00	Ser	Asn
EDX74960	*Coleofasciculus chthonoplastes*	280	38.78	53.40	Asp	Ile
ASR47350	*Paenibacillus kribbensis*	299	38.54	50.96	Asp	Thr
EWS96050	*Streptomyces filamentosus*	293	37.82	50.96	Asp	Val
SHF36578	*Seinonella peptonophila*	285	37.62	52.15	Asp	His
OKP91661	*Paenibacillus sp.*	287	37.17	49.67	Asp	Val
EDY63080	*Streptomyces pristinaespiralis*	327	36.71	46.24	Asp	Thr
RGJ04542	*Hungatella hathewayi*	276	35.55	47.84	Asp	Cys

KZE50427	*Brevibacillus parabrevis*	281	35.50	51.47	Asp	Asn
OON56752	*Flavobacterium sp.*	272	33.78	48.43	Asp	Asn
ABQ03789	*Flavobacterium johnsoniae*	272	33.14	48.16	Asp	Asn

Supplementary Table 2. Oligonucleotide primers used in the study.

Name	Sequence: 5' – 3'
	Cloning for recombinant protein expression
DrPARG-F	GGGATCCCATATGAACCGCAAA
DrPARG-R	GAATTCTCGAGTCAGGAGGTAGATGAGGGC
T267R-F	TCAGCACCCGAGGCTCGGCGCGT
T267R-R	ACGCGCCGAGCCTCGGGTGCTGA
T267K-F	TCAGCACCCGAAACTCGGCGCGTTT
T267K-R	AAACGCGCCGAGTTTCGGGTGCTGA
E112A-F	GGCGCGCAGGCCCAGGAAGCAGACCTGTGCCGTGGCAGT
E112A-R	ACTGCCACGGCACAGGTCTGCTTCCTGGGCCTGCGCGCC
HsPARP1-F	AATTCATATGGCGGAGTCTTCGGATAAG
HsPARP1-R	AATTCTCGAGTTACCACAGGGAGGTCTTAA
HsPARP10 CatD-F	CGGCATATGAACAACCTGGAGCGTCTGGCA
HsPARP10 CatD-R	AATTCTCGAGTTAAGTGTCTGGGGAGCGGCC
	Generation of Δ*parg* strain
P1	CTCTACTCTACGCAGCAGTGATCC
P2	GTTCCAGATAGTCGGCGGTGTC
P3	ACAGACAGCGCTTAGAAAAACTCATCGAGCATCAAATG
P4	TTCTAGGGGCCCCGCCAAGCTCGCGAGGCC
	RAPD analysis
AS-10	CCCGTCTACC

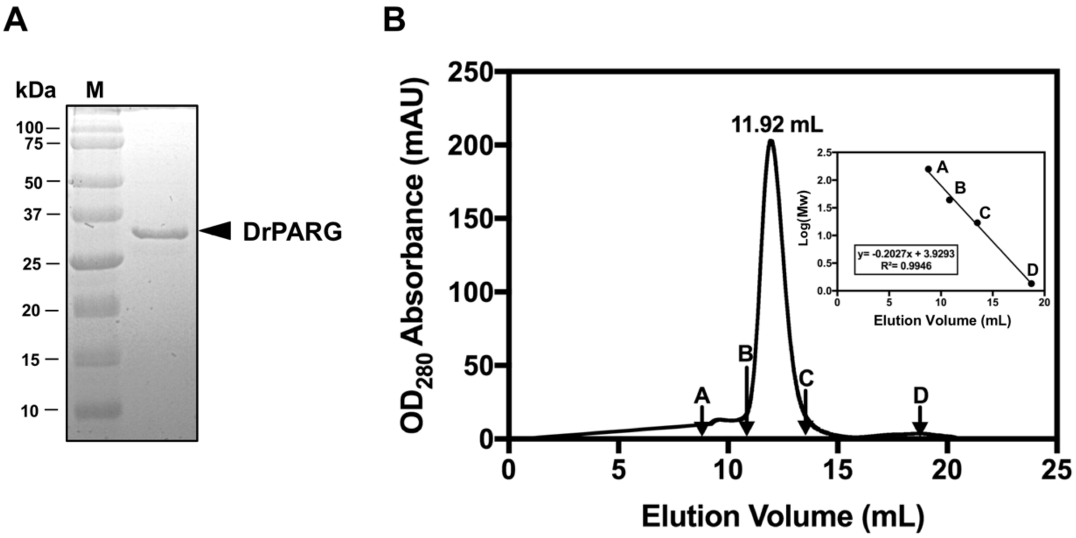

Supplementary Figure 1. Gel filtration chromatography analysis of DrPARG.

(A) SDS-PAGE analysis of purified DrPARG. (B) Gel filtration chromatography analysis of DrPARG. The inset shows the logarithm of molecular weight of markers (A, bovine gamma globulin, 158 kDa; B, chicken ovalbumin, 44 kDa; C, horse myoglobin, 17 kDa; D, vitamin B_{12}, 1.35 kDa) plotted against the elution volume (mL) and the standard curve equation is $y = -0.2027x + 3.9293$ with $R^2 = 0.9946$. The calculated molecular weight of DrPARG (≈ 32 kDa) suggests that it is a monomer in solution. Source data are provided as a Source Data file.

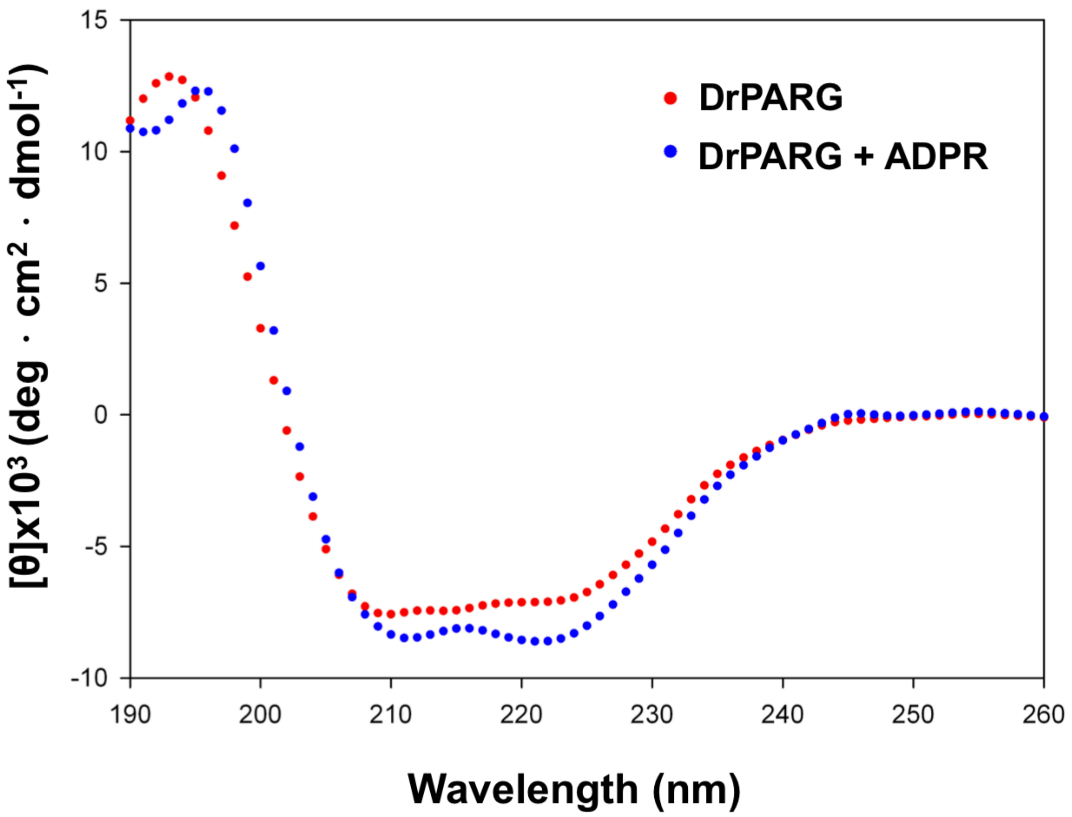

Supplementary Figure 2. Folding of DrPARG in the presence or absence of ADP-ribose.

The CD spectra were recorded at 10°C with 10 µM DrPARG in CD buffer (20 mM phosphate buffer, pH 7.5) from 260 to 190 nm. The scatterplot shows the DrPARG with or without pre-incubation of 30 µM ADP-ribose, in blue and red circles, respectively.

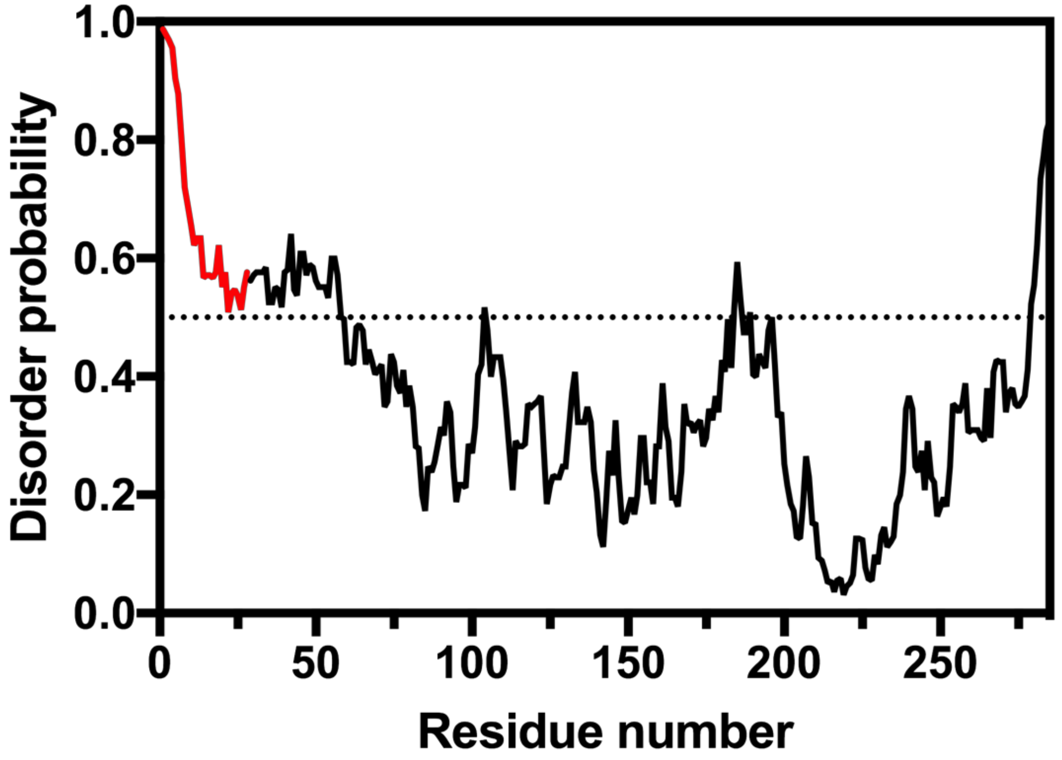

Supplementary Figure 3. Prediction of intrinsically disordered region of DrPARG. The residues not well defined in the electron density maps of ADP-ribose bound PARG structure (red) are located in the N-terminal disordered region of the protein. Disorder probability of DrPARG was calculated by IUpred software.

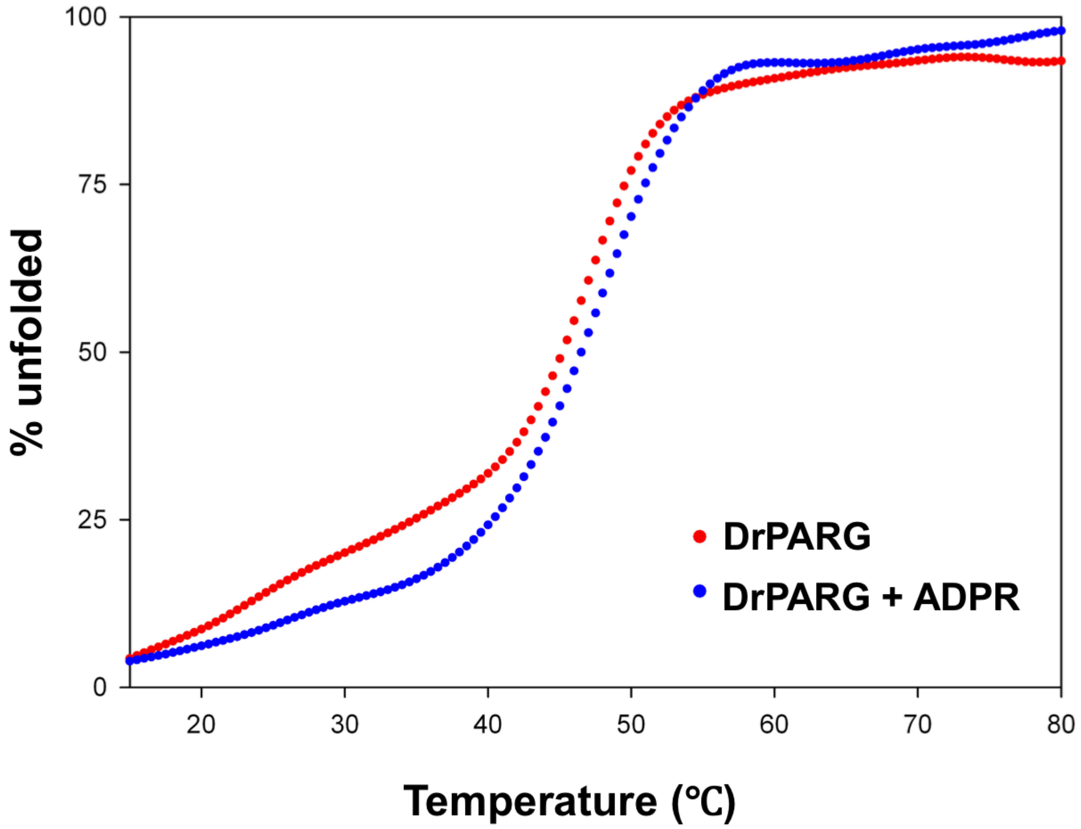

Supplementary Figure 4. Thermal denaturation of DrPARG in the presence or absence of ADP-ribose.

CD spectra were recorded at 220 nm with 10 μM DrPARG in CD buffer (20 mM phosphate buffer, pH 7.5) from 10°C to 95°C. The scatterplot shows the DrPARG with or without pre-incubation of 30 μM ADP-ribose, in blue and red circles, respectively.

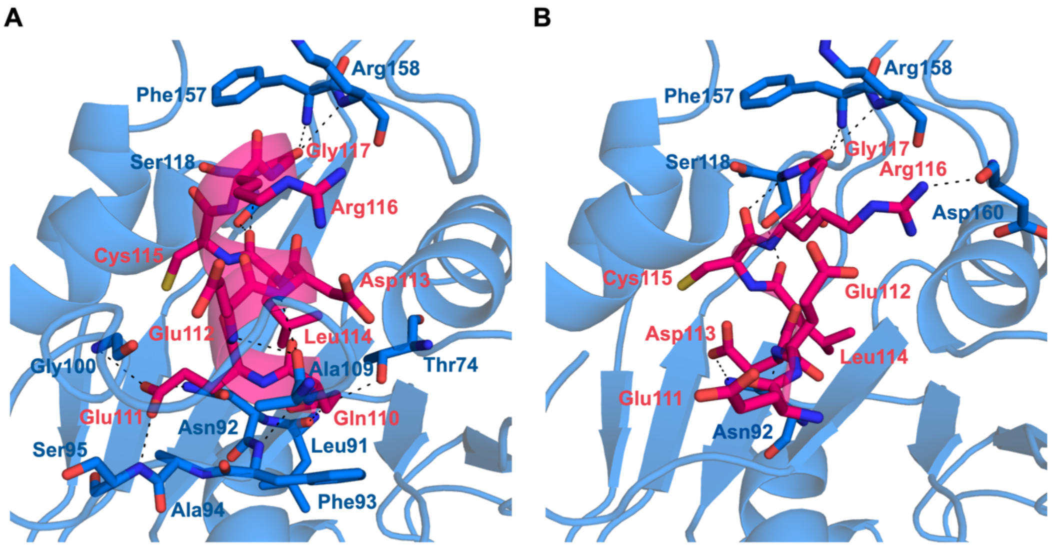

Supplementary Figure 5. Comparison of hydrogen bonding network for the region forming the inducible helix of DrPARG.

The hydrogen bonding network for (A) α4’ helix in ADP-ribose bound form and (B) its equivalent region in the apo form interacting with the protein. Residues involved in hydrogen bond formation are labeled and shown as sticks with carbons colored individually (pink for α4’ helix and blue for protein). Oxygen, nitrogen, and sulfur are colored red, blue, and yellow, respectively. Hydrogen bonds are shown as dashed lines.

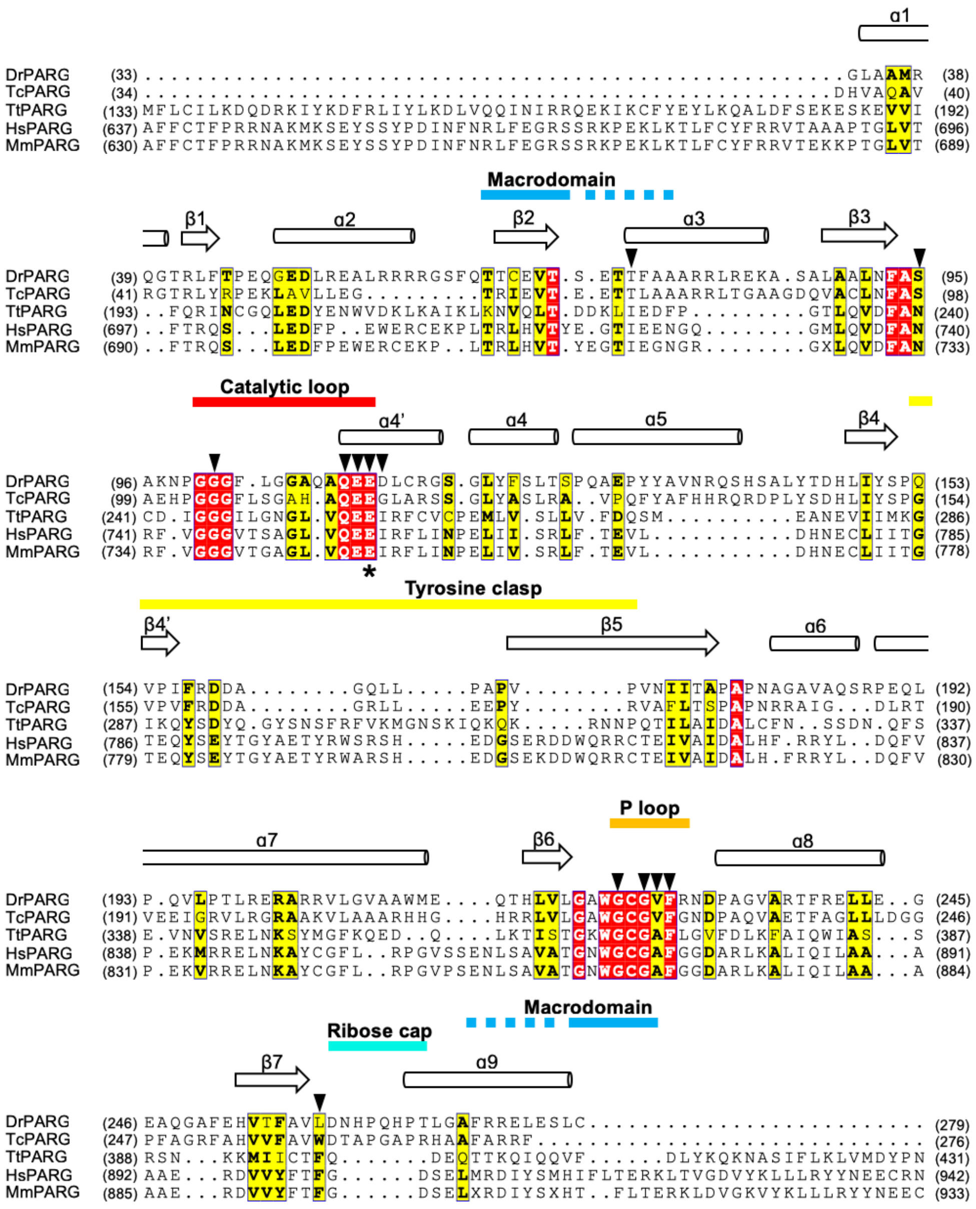

Supplementary Figure 6. Structure based alignment of PARG sequences.

The sequence of DrPARG was aligned with those of previously reported canonical and bacterial PARGs. (Dr, *Deinococcus radiodurans*, PDB code 5ZDB; Tc, *Thermomonospora curvata*, PDB code 3SIG; Tt, *Tetrahymena thermophila*, PDB code 4EPP; Hs, *Homo sapiens*, PDB code 4B1H; Mm, *Mus musculus*, PDB code 4NA0). Consensus amino acids among PARGs with similarity score >0.7 are framed in yellow. Identical amino acids are in white and framed in red. Secondary structures of DrPARG are depicted on the top of the alignment with arrows for β strands and cylinders for α helices. Conserved motifs and the N- and C-terminal extents of the macrodomain core are labelled on the top of the sequence and indicated by colored lines. Key residues in the ADP-ribose binding pocket are labelled with black triangles and the conserved catalytical glutamate is labelled with an asterisk.

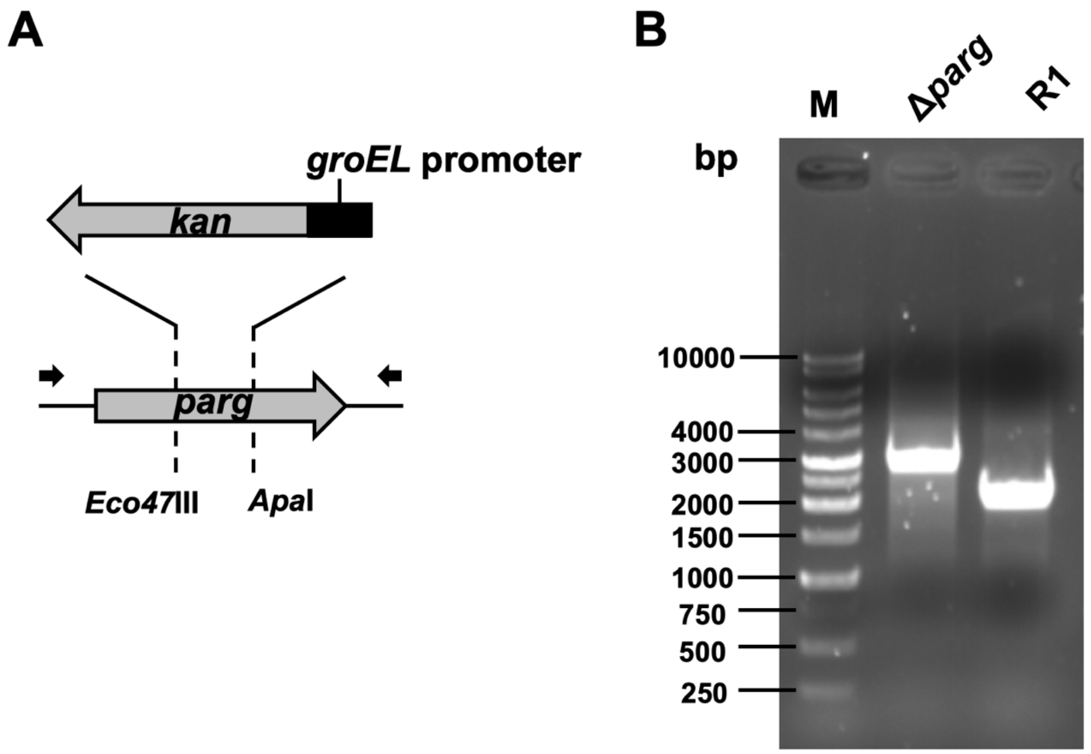

Supplementary Figure 7. Schematic representation and validation of deletion in *D. radiodurans parg* gene.

(A) Schematic diagram of the allele replacement event in *parg* gene. Black arrowheads indicate the position of diagnostic primers used for PCR validation. (B) PCR analysis of genomic DNA from R1 and Δ*parg* strains using diagnostic primers. Source data are provided as a Source Data file.

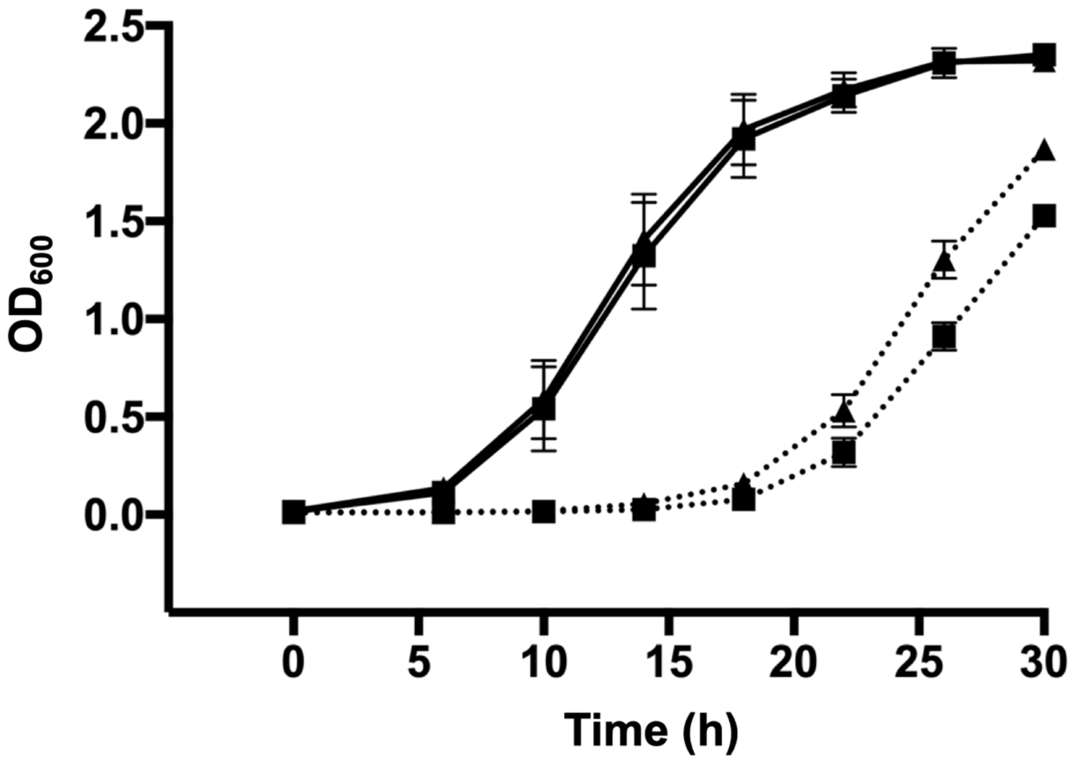

Supplementary Figure 8. Δ*parg* is more susceptible to UV radiation than R1. Growth curves (monitored by OD_{600}) are represented by black triangles and squares for R1 wild-type and Δ*parg* strains, respectively. Non-irradiated and irradiated cells are represented by solid and dashed lines, respectively. Irradiated cultures (OD ≈ 0.8) were exposed to a germicidal UVC lamp (254 nm) at 0.3 $J/m^2/sec$ before 1:100 dilution into TGY media. Data are plotted as mean ± SEM (n = 3 independent experiments). Source data are provided as a Source Data file.

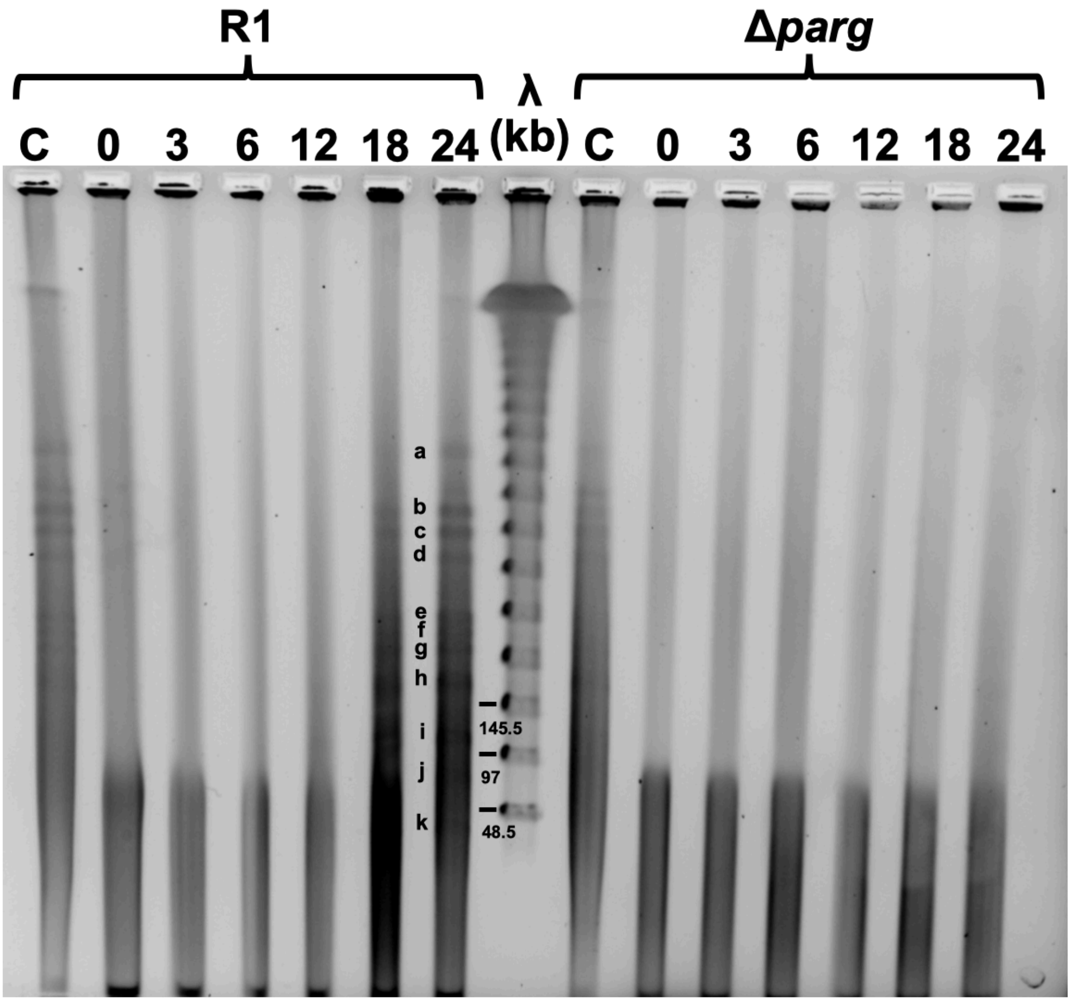

Supplementary Figure 9. Genome recovery of R1 and Δ*parg* strains analyzed by pulsed-field gel electrophoresis (PFGE).

R1 and Δ*parg* cells irradiated with a 900 J/m^2 UVC dose were placed in TGY medium for recovery over a 24 h time course and PFGE samples were collected at indicated time points. λ, lambda DNA ladder. C, non-irradiated culture. a-k, resolvable *Not*I junction fragments. Source data are provided as a Source Data file.

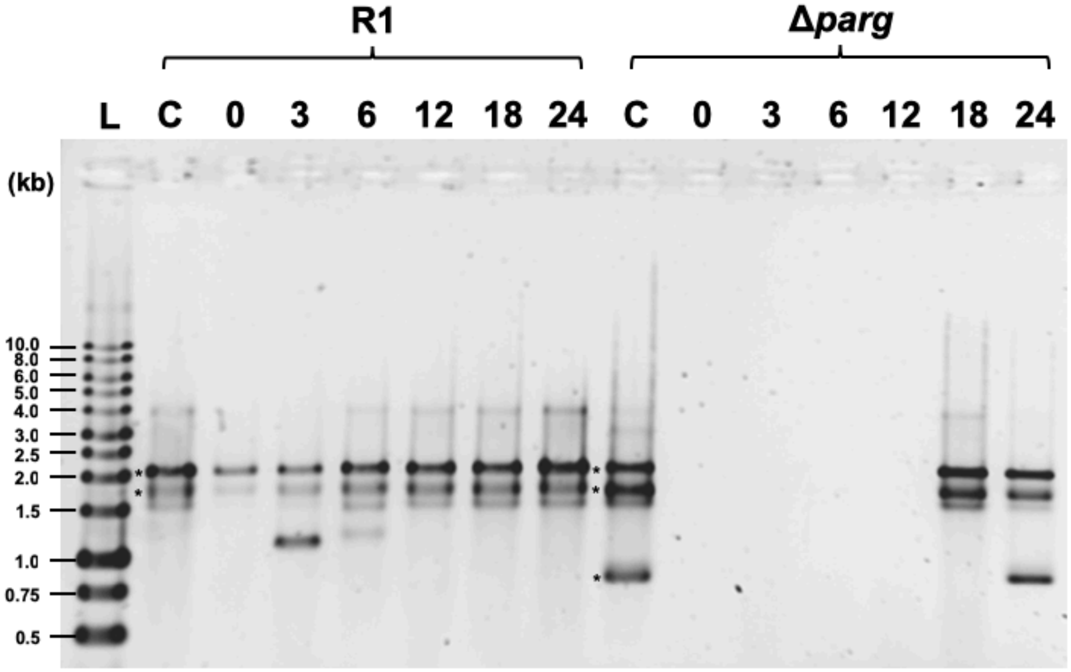

Supplementary Figure 10. Restoration of genome integrity in R1 and Δ*parg* strains analyzed by random-amplified polymorphic DNA (RAPD).

R1 and Δ*parg* cells irradiated with a 900 J/m^2 UVC dose were placed in TGY medium for recovery over a 24 h time course and RAPD samples were collected at indicated time points. L, DNA ladder. C, non-irradiated culture. The PCR products of high intensity generated from non-irradiated cultures of R1 and Δ*parg* were labelled by asterisks. Source data are provided as a Source Data file.

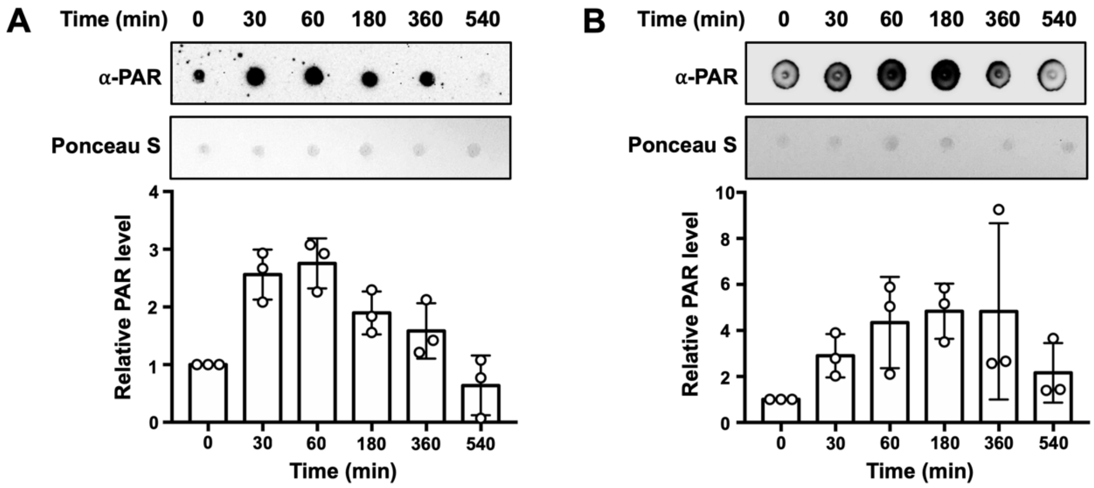

Supplementary Figure 11. The dynamics of PAR level in R1 and Δ*parg* cells receiving UV irradiation.

PAR level was monitored by dot blotting in non-irradiated cells (time = 0) and cells harvested at 30, 60, 180, 360, and 540 min after exposure to UV from (A) R1 and (B) Δ*parg* strains. Ponceau Red staining was the loading control. PAR levels were quantified by using Image J and plotted against recovery time as mean ± SEM relative to non-irradiated cells, with PAR levels set to 1 (n = 3 independent experiments). Source data are provided as a Source Data file.

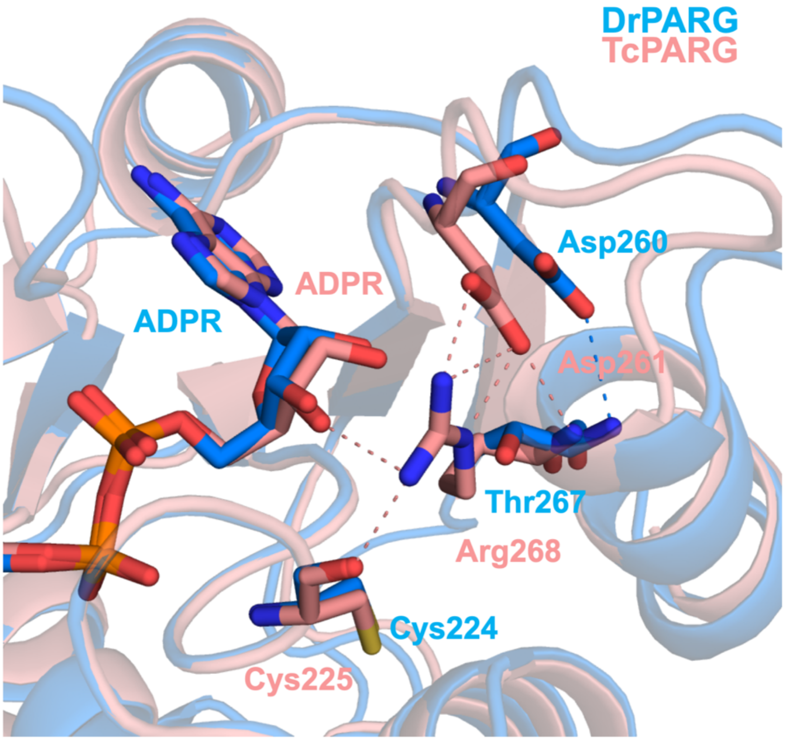

Supplementary Figure 12. Comparison of the interaction network for Arg-268 of TcPARG and corresponding residue of DrPARG.

Superposition of ADP-ribose bound structures of TcPARG and DrPARG is shown as cartoon models in salmon and blue, respectively. Key residues and ADP-ribose are labeled and shown as sticks with carbon in the same color as in the cartoon representation. In stick representations, nitrogen is in blue, oxygen is red, phosphorus is orange, and sulfur is yellow. Hydrogen bonds are shown as dashed lines.

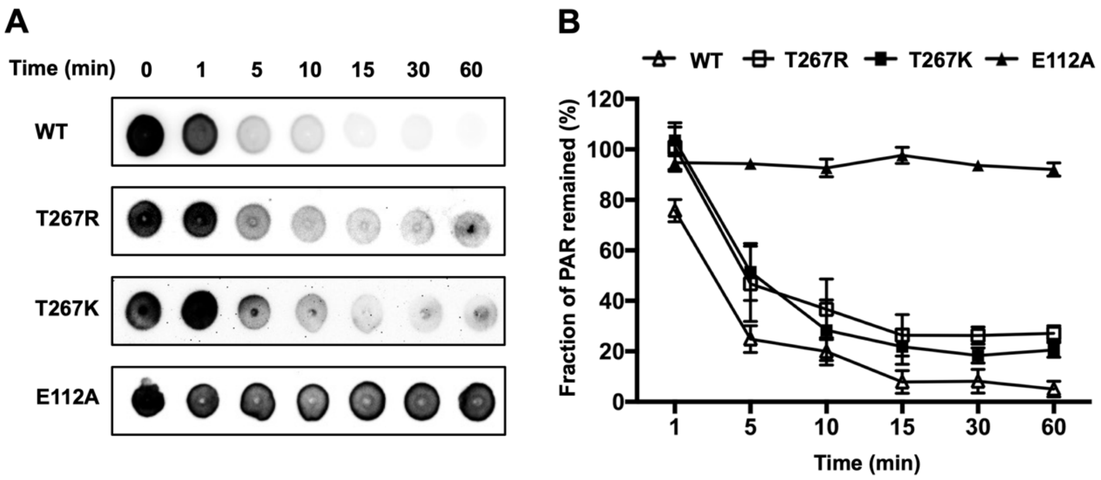

Supplementary Figure 13. PAR cleavage activity of wild-type and mutant PARG. (A) The reaction of wild-type and mutant DrPARG treated with or without (time = 0) automodified PARP1 was stopped at different times and PAR was assayed by dot blotting. (B) Fractions of PAR remaining were quantified by using Image J software and plotted against time as mean ± SEM relative to untreated sample, with PAR levels set to 100% (n = 3 independent experiments). WT and T267R are represented as open triangle and square, respectively. T267K and E112A are represented as solid square and triangle, respectively. Source data are provided as a Source Data file.

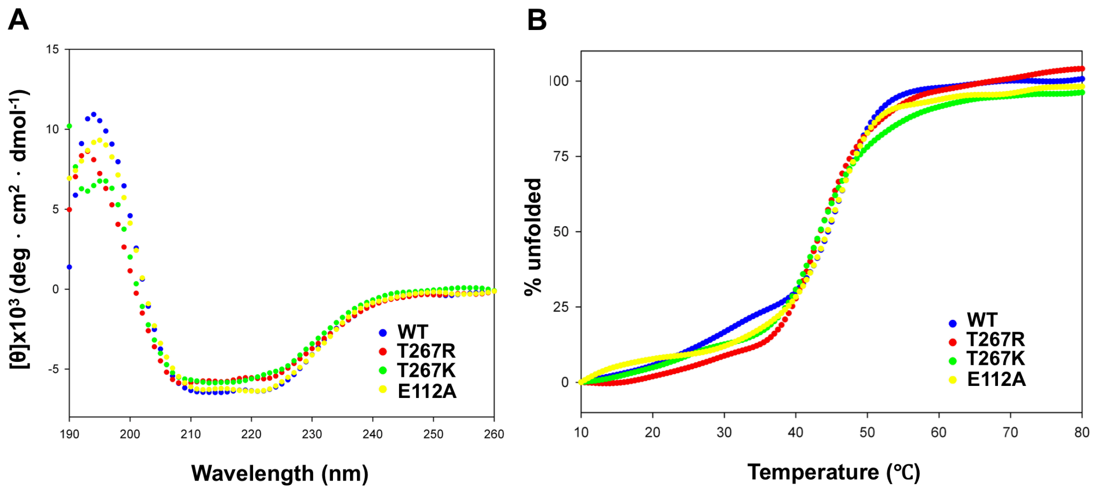

Supplementary Figure 14. Folding and thermal denaturation of wild-type and mutant DrPARG analyzed by circular dichroism (CD).

(A) Folding of wild-type and mutant DrPARG assayed by CD spectra recorded at 10 °C with 10 μM purified protein in CD buffer (20 mM phosphate buffer, pH 7.5) from 260 to 190 nm. (B) Thermal denaturation of wild-type and mutant DrPARG assayed by CD spectra recorded at 220 nm with 10 μM purified protein in CD buffer (20 mM phosphate buffer, pH 7.5) from 10 to 95 °C. The scatterplots show the wild-type, T267R, T267K, and E112A mutant DrPARG in blue, red, green, and yellow, respectively. The calculated Tm for the wild-type, T267R, T267K, and E112A mutant DrPARG was 44.50, 43.44, 43.38 and 44.41 °C, respectively. WT, T267R, T267K, and E112A are represented as blue, red, green, and yellow circles, respectively.

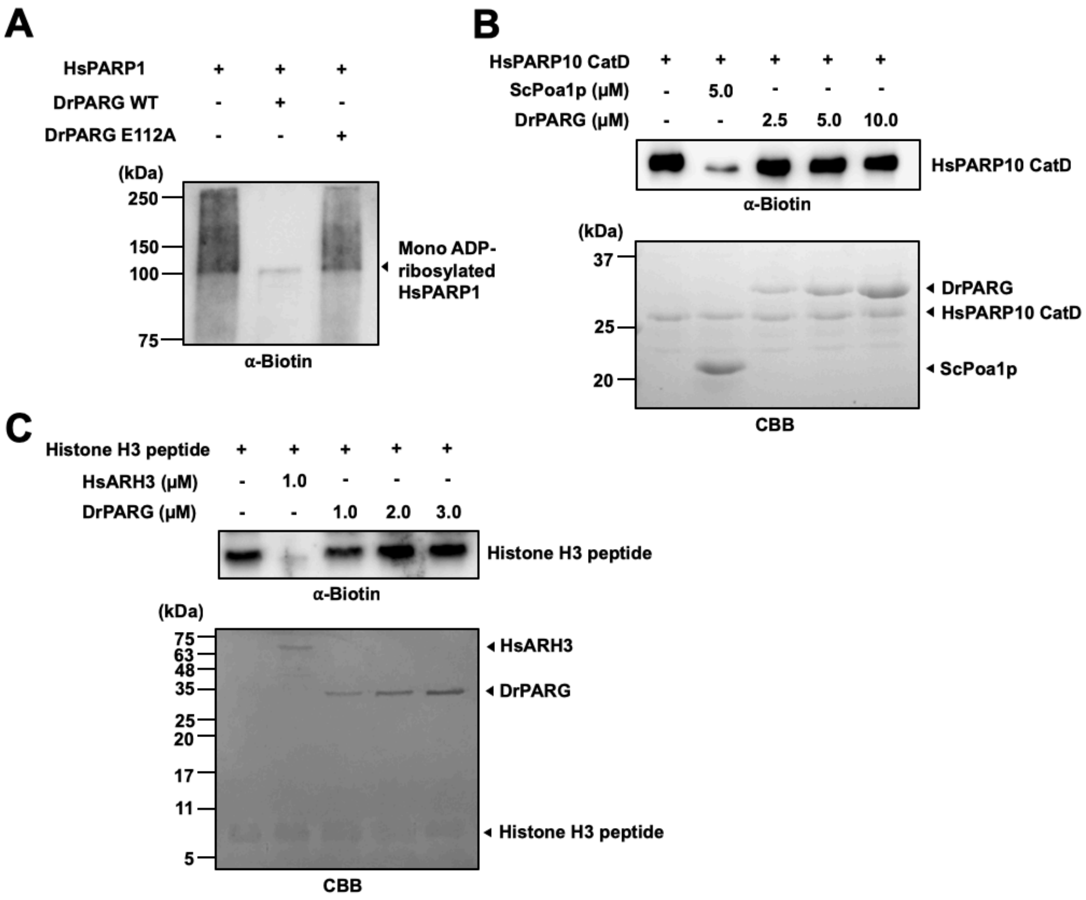

Supplementary Figure 15. DrPARG is incapable of processing glutamate- and serine-linked mono ADP-ribosylation.

(A) Human PARP1 (HsPARP1) was poly ADP-ribosylated using biotinylated NAD^+ and incubated with WT and E112 DrPARG. Hydrolysis of PAR was assayed by western blot using biotin antibody. DrPARG processes poly ADP-ribosylation but not mono ADP-ribosylation of HsPARP1. (B) Human PARP10 catalytic domain (HsPARP10 CatD) was mono ADP-ribosylated using biotinylated NAD^+. Hydrolysis of mono ADP-ribosylation was assayed by western blot using biotin antibody. The reaction incubated with Poa1p from the budding yeast (ScPoa1p) was used as a positive control of hydrolysis of glutamate-linked mono ADP-ribosylation. The gel was stained by Coomassie Brilliant Blue (CBB) as a loading control. (C) Human histone H3 peptide (a.a. 1-21) was mono ADP-ribosylated using biotinylated NAD^+. Hydrolysis of mono ADP-ribosylation was assayed by western blot using biotin antibody. The reaction incubated with human ARH3 (HsARH3) was used as a positive control of hydrolysis of serine-linked mono ADP-ribosylation. The gel was stained by Coomassie Brilliant Blue (CBB) as a loading control. Source data are provided as a Source Data file.

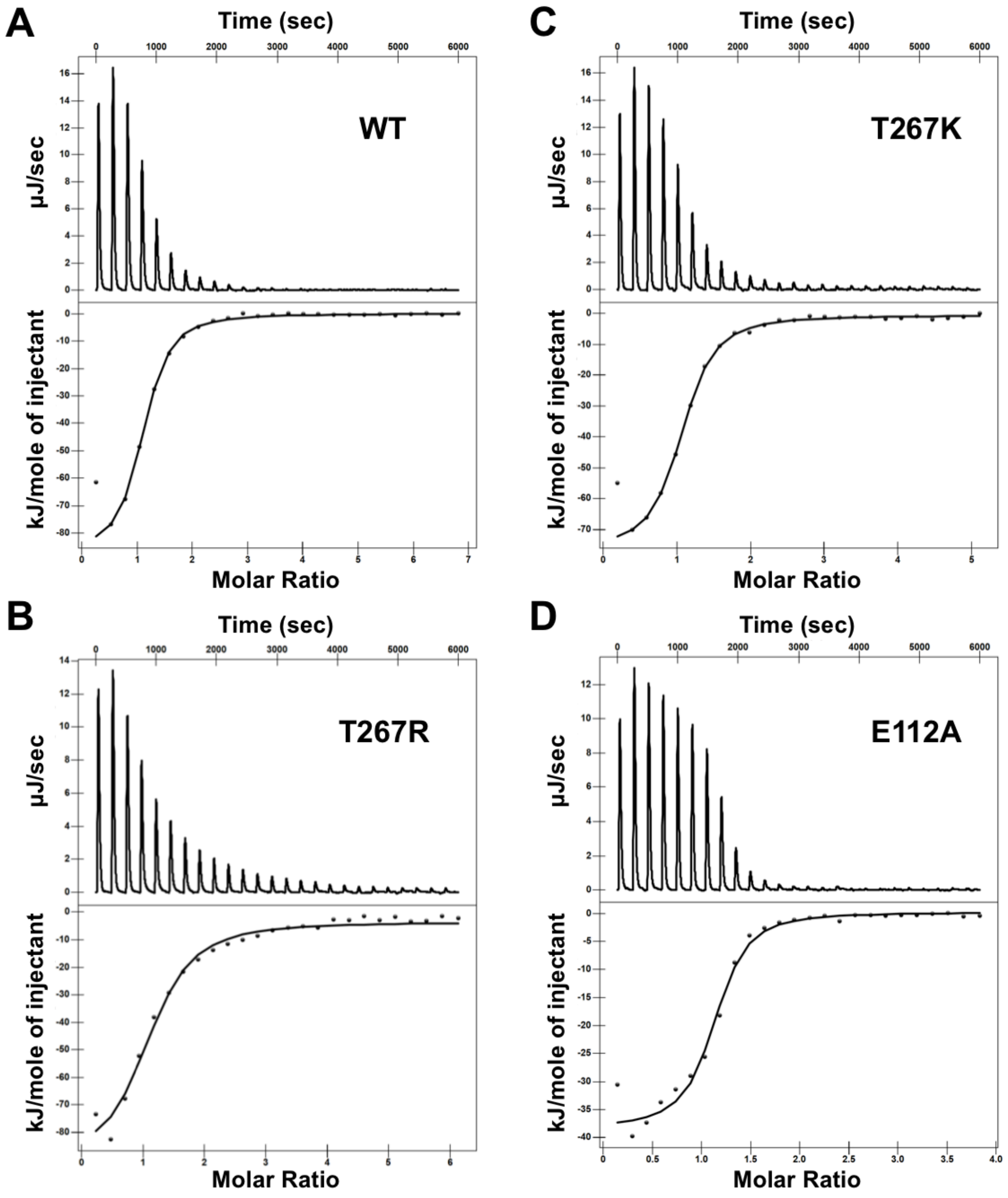

Supplementary Figure 16. Representative isotherms from isothermal titration calorimetry (ITC) analysis of ADP-ribose binding to wild-type and mutant DrPARG.

ITC analysis of (A) WT, (B) T267R, (C) T267K, and (D) E112A mutant DrPARG. Upper panel, raw data in μJ/s versus time showing heat release on injection of 1.5 – 3.0 mM ADP-ribose into a 1030 μL cell containing 0.075 – 0.150 mM protein. Lower panel, integration of raw data yielding the heat per mole versus molar ratio.

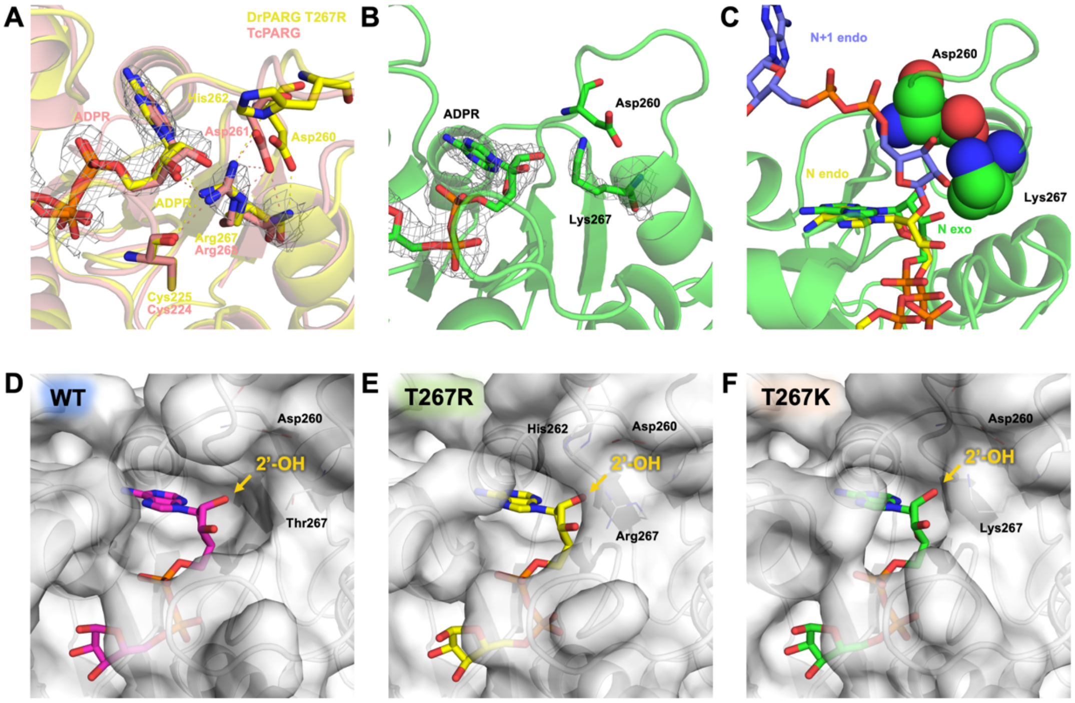

Supplementary Figure 17. Structural analysis of DrPARG (T267R and T267K) mutants in complex with ADP-ribose.

(A) Superposition of ADP-ribose bound structures of TcPARG and DrPARG T267R mutant. The $2F_o$-F_c difference map of ADP-ribose and arginine, contoured at 1σ, was calculated at 2.60-Å resolution from a model with the ligand and residue omitted. Key residues and ADP-ribose molecule are labeled and shown as sticks. Hydrogen bonds are shown as dashed lines. (B) Structure of DrPARG T267K mutant. The $2F_o$-F_c difference map of ADP-ribose and lysine, contoured at 1σ, was calculated at 2.50-Å resolution from a model with the ligand and residue omitted. Key residues and ADP-ribose molecule are labeled and shown as sticks. Hydrogen bonds are shown as dashed lines. (C) An overlay of the modeled endo-glycohydrolase PAR (carbons of n+1 and n units are colored distinctly) with the structure of DrPARG T267K mutant. Residues implicated in steric hindrance with the n+1 ADP-ribose unit are shown as spheres. For each labeled ADP-ribose/residue in TcPARG and DrPARG mutant structures, carbons are colored distinctly according to their individual cartoon models. For atoms in stick representations, nitrogen, oxygen, phosphorous and sulfur are colored blue, red, orange, and yellow, respectively. Solvent accessibility of bound ADP-ribose in (D) WT, (E) T267R, and (F) T267K DrPARG. ADP-ribose is shown as sticks with carbons colored individually among the three structures. Selected residues are labeled. The hydroxyl of ribose' with n+1 linkage point is labeled and indicated by arrowheads.

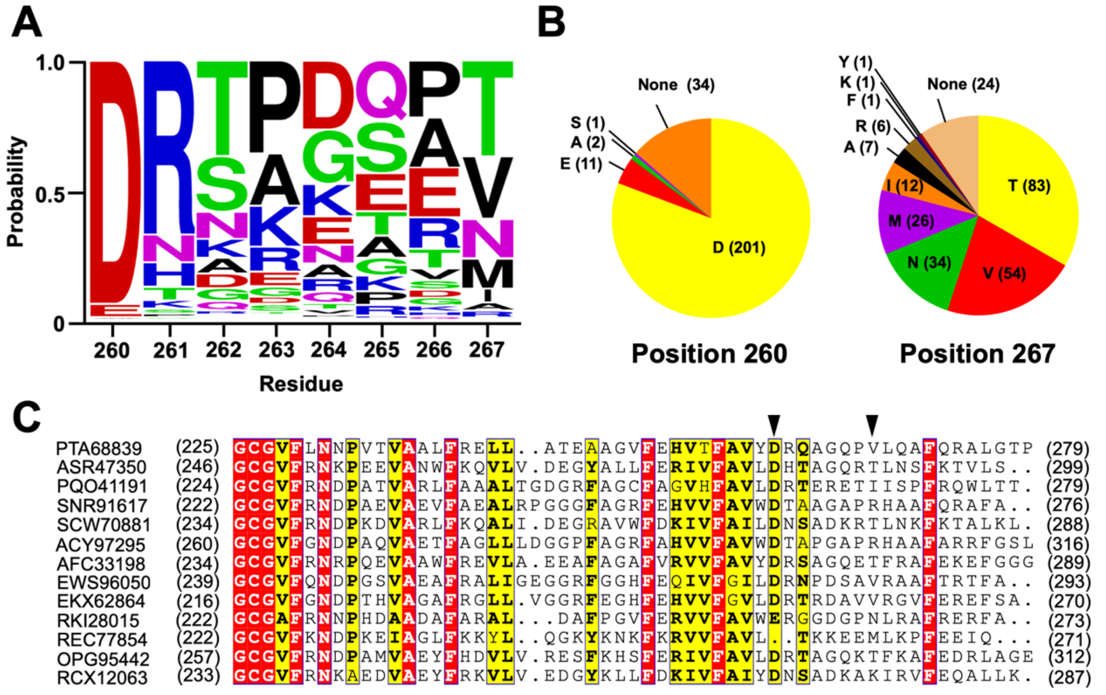

Supplementary Figure 18. Analysis of sequences from BLAST hits of DrPARG bearing the PARG signature motif.

(A) Sequence conservation between position 260 and 267 corresponding to DrPARG in aligned BLAST hits. (B) Pie diagrams depicting the occurrence of amino acids in the position 260 and 267. (C) Sequence alignment of the C-terminal regions of selected BLAST hits of DrPARG. Consensus amino acids with similarity score >0.7 are framed in yellow. Identical amino acids are in white and framed in red. Position 260 and 267 corresponding to DrPARG are labelled by black triangles on the top of the sequences. Protein accession numbers are shown on the left of the alignment.

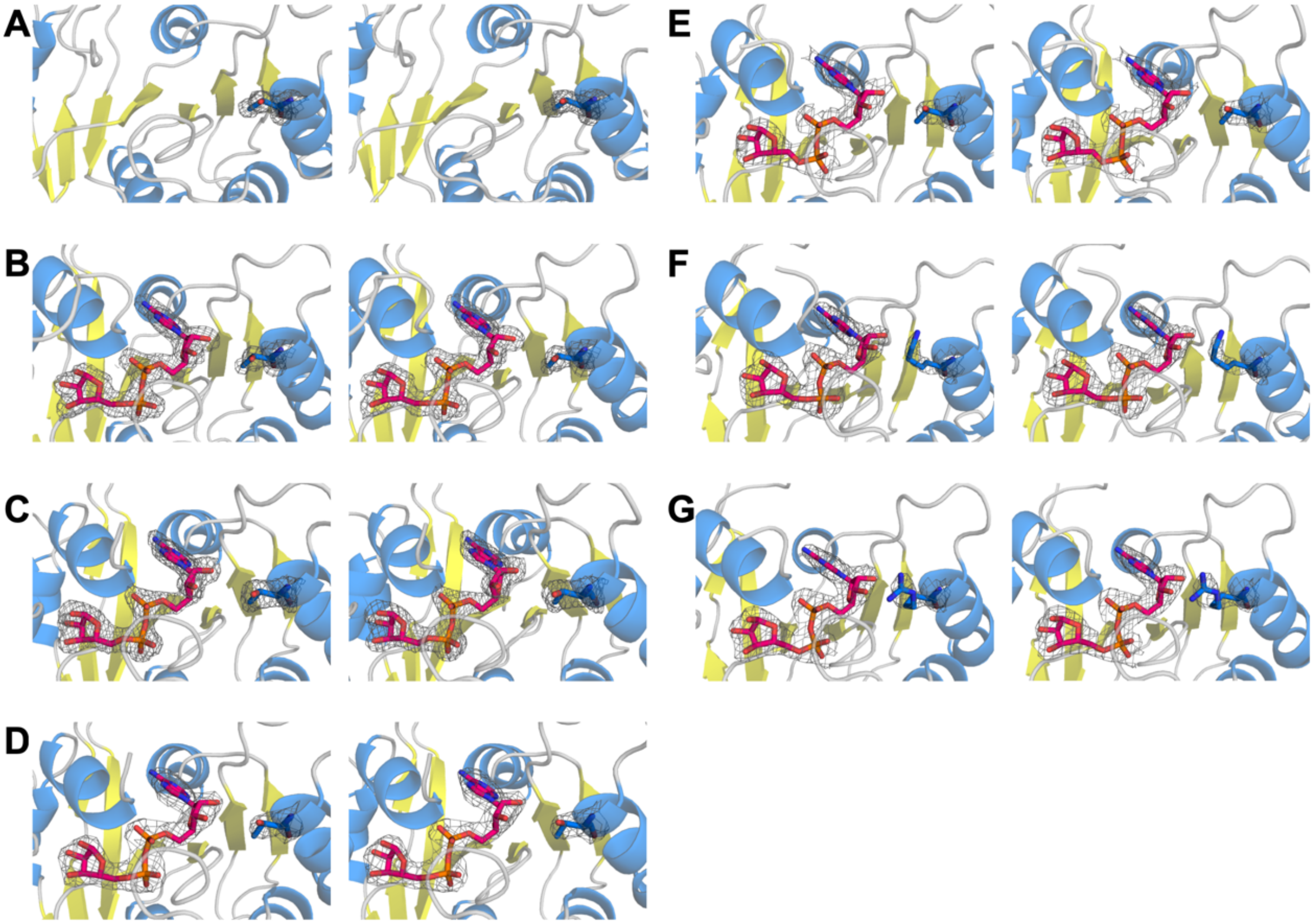

Supplementary Figure 19. Electron density maps in the DrPARG structures.

Stereograms of the $2F_o$-F_c difference map, contoured at 1σ, for equivalent residue in the position 267 or ADP-ribose in (A) 5ZDA (calculated at 1.55 Å resolution), (B) 5ZDB (calculated at 1.97 Å resolution), (C) 5ZDC (calculated at 1.98 Å resolution), (D) 5ZDD (calculated at 2.72 Å resolution), (E) 5ZDE (calculated at 2.81 Å resolution), (F) 5ZDF (calculated at 2.50 Å resolution), and (G) 5ZDG (calculated at 2.60 Å resolution). The color of protein and ADP-ribose is the same as in Figure 1B. ADP-ribose and residue in the position 267 are shown as sticks.
